# Analysis of dentin wear and biological properties promoted by experimental inoffice desensitizing materials

**DOI:** 10.1186/s12903-024-04373-9

**Published:** 2024-05-25

**Authors:** Fernanda de Souza Silva Ramos, Laryssa de Castro Oliveira, Larissa Albertinazzi, Sávio José Cardoso Bezerra, Vanessa Rodrigues dos Santos, Tais Scaramucci, Cristiane Duque, Bernhard Ganss, Marina Trevelin Souza, Juliano Pelim Pessan, Ticiane Cestari Fagundes

**Affiliations:** 1 Department of Preventive and Restorative Dentistry, School of Dentistry, São Paulo State University (UNESP), Araçatuba, São Paulo, SP Brazil; 2 Department of Restorative Dentistry, University of São Paulo (USP), School of Dentistry, São Paulo, SP Brazil; 3 Faculty of Dentistry, Institute of Biomedical Engineering, University of Toronto, Toronto, Ontário Canada; 4 Vitreous Materials Laboratory, Department of Materials Engineering, Federal University of São Carlos, São Carlos, São Paulo, SP Brazil

**Keywords:** Cell biology, Cytotoxicity tests, Dental abrasion, Desensitizing agents, Dental erosion, Dentin sensitivity

## Abstract

**Background:**

This study aimed to evaluate dentin wear and biological performance of desensitizing materials.

**Methods:**

Seventy bovine root dentin blocks were sectioned. Half of the surface of each specimen was untreated (control) and the other half was immersed in EDTA and treated with the following desensitizing materials: placebo varnish (PLA), fluoride varnish (FLU), sodium fluoride (NaF) varnish + sodium trimetaphosphate (TMP), universal adhesive (SBU), S-PRG varnish (SPRG), biosilicate (BIOS), and amelotin solution (AMTN). After application, the specimens were submitted to an erosive-abrasive challenge and the wear analyzed by optical profilometer. Serial dilutions of extracts obtained from the culture medium containing discs impregnated with those desensitizers were applied on fibroblasts and odontoblasts-like cells cultures. Cytotoxicity and production of total protein (TP) by colorimetric assays were determined after 24 h. Data were statistically analyzed using Kruskal-Wallis, Dunn’s, One-way ANOVA and Tukey tests (*p* ≤ 0.05).

**Results:**

No dentin wear was observed only for SBU. The lowest dentin wear was observed for AMTN and TMP. Cell viability was significantly reduced after treatment with undiluted extracts of PLA, FLU, TMP and SBU in fibroblasts and TMP and SBU in odontoblast-like cells. SPRG, BIOS and AMTN were cytocompatible at all dilutions tested. Considering TP results, no statistical difference was observed among the groups and high levels for TP were observed after TMP and FLU treatments.

**Conclusions:**

Universal adhesive system may protect dentin with opened tubules from wear after challenge. Extracts of adhesive and fluoride varnishes presented cytotoxic mainly on fibroblasts. The enamel protein may be a future alternative to treat dentin with opened tubules because it may cause low wear under erosive-abrasive challenge with low cytotoxic effects.

**Supplementary Information:**

The online version contains supplementary material available at 10.1186/s12903-024-04373-9.

## Background

Erosive tooth wear is a dental clinical condition with global prevalence estimated between 20 and 45% in permanent teeth [1]. Erosive tooth wear is a gradual loss of dental hard tissues with multifactorial etiology involving chemical, biological and behavioral factors. The exposure of dentinal tubules by erosive tooth wear is probably the major predictor of dentin hypersensitivity that is considered one of the most common complaints from patients [2–4]. Dentin hypersensitivity is characterized as a short, sharp pain that arises from exposed dentin in the cervical region, caused by abrasion, erosion, and/or abfraction [5]. Teeth with dentin hypersensitivity should be treated considering the related-risk factors and severity [6, 7].

Since the mechanisms of dentin hypersensitivity is still unclear, some theories have been described in the literature [8]. (1) hydrodynamic theory, (2) direct innervation of dentinal tubules, (3) neuroplasticity and sensitization of nociceptors, (4) odontoblasts serving as sensory receptors, and (5) algoneurons. While the hydrodynamic theory has been the most widely accepted concept, recent research has raised questions that require further investigation. Recent interest has focused on the role of mechanosensitive ion channels, sodium channels, and adenosine triphosphate activation in tooth pain as well as the function of odontoblasts as primary sensory cells or in collaboration with other signals and neurotransmission [8]. Disrupting these mechanisms may lead to effective treatments for pulpal pain. These mechanisms may also be influenced by pulpal responses to tissue injury such as neuronal sprouting and peripheral sensitization [8]. A better understanding of these mechanisms may contribute to the development of therapeutic drugs that target them.

Despite the wide range of commercially available products for the treatment, there is no “gold standard” therapy for dentin hypersensitivity [5]. The preventive approaches recommended for erosive tooth wear are based on the prevention of erosive acids attacks to the teeth caused by both extrinsic and intrinsic factors [6]. In addition, it is recommended the protection of the tooth structure with dental materials to create an extra mechanical barrier against the erosive acids [6]. In the presence of dentin hypersensitivity, strategies of treatment have been developed to modify nociceptive response to promote dentinal tubules occlusion [3]. In this context, fluoride-based varnishes, photocured agents and experimental materials have been used to decrease the DH, by means of the tubular occlusion [9, 10].

Fluoride varnishes represent the most used treatment for dentin hypersensitivity due to the formation of calcium fluoride precipitates [11, 12]. However, a reduction in dentin hypersensitivity up to the first month of treatment have been reported, which is very reduced after three months of application, because those precipitates are not resistant in the oral environment conditions [13]. The addition of inorganic phosphate salts has been proposed as a method to increase the resistance of fluoride varnishes overtime [14, 15]. Previous studies have shown the ability of sodium trimetaphosphate (TMP) in protecting the collagen matrix and promoting the deposit of calcium phosphate-apatite, protecting the dentin against erosion by tubules occlusion [10, 14, 15].

Among the photocured agents, universal adhesive promotes a desensitizing effect by dentin tubules sealing by hybrid layer formation, which is able to neutralize the hydrodynamic mechanism of hypersensitivity [16]. Recently, an innovative material combining a light-curing fluoride varnish with multifunctional pre-reacted glass particles, S-PRG, has promoted dentin tubules occlusion through bioactive technology [9].

Experimental solutions with bioactive ceramics have also shown precipitation of calcium phosphate and hydroxyapatite formation; promoting the occlusion of the dentinal tubules, and preventing dentin demineralization [10, 17, 18]. Another innovative material is a protein expressed during the maturation phase of tooth enamel formation (amelotin) which is able to bond and form protein complexes, promoting calcium phosphate precipitation, dose-dependent hydroxyapatite formation and collagen matrix mineralization [19–21]. The amelotin was considered as a novel factor produced by ameloblasts that plays a critical role in the formation of dental enamel [21].

Since desensitizers are applied in erosive highly sensitive dentin with exposed tubules, the evaluation of dentin wear and cytotoxicity becomes a requirement before their indication [22–24]. However, studies have measured dentin wear treated with fluoride varnishes submitted to erosive-abrasive cycling [24], there is a lack of studies evaluating the wear, by optical profilometry, of experimental desensitizers on previously eroded dentin. Few studies have also shown that fluoride varnishes, bioactive ceramics and universal adhesive present low cytotoxicity [22, 23, 25, 26]. Nevertheless, there is a lack of information about biological properties of recently launched and experimental materials that may be indicated to dentin hypersensitivity therapies, mainly with respect to the use of amelotin protein in eroded dentin and its biological properties. Amelotin is a novel factor produced by ameloblasts that plays a critical role in the formation of dental enamel; however, the literature presents few, but promising results, about its application in eroded dentin when dentin permeability and tubule occlusion were evaluated [10].

In this context, the present study aimed to evaluate the effects of different in-office desensitizing agents on dentin wear protection and biological properties, essentially to study the viability about the use of amelotin protein to treat dentin with opened tubules. The null hypothesis tested were: (1) there would be no difference among the materials in the protection of dentin erosive wear after erosive-abrasive challenge; and (2) there would be no difference in the among the material considering their effect on the viability and protein production by fibroblasts and odontoblast-like cells.

## Methods

This research was conducted after approval by the Local Ethics Committee on Animal Experiments (Process #00.418–2020).

### Study design

This study tested 7 desensitizing agents, in an erosion-toothbrushing cycling model of 5 days, using seventy bovine dentin specimens (*n* = 10). The response variable was dentin wear (µm), using an optical profilometer. Biological properties were also analyzed by cytotoxicity and total protein production, determined for two different cells lines (fibroblasts and odontoblast-like cells) and colorimetric assays (resazurin and Lowry methods), using different extract dilutions in 24 h. All experiments of biologic properties were performed in duplicate in two independent experiments (*n* = 6) [28].

**Fig. 1:**
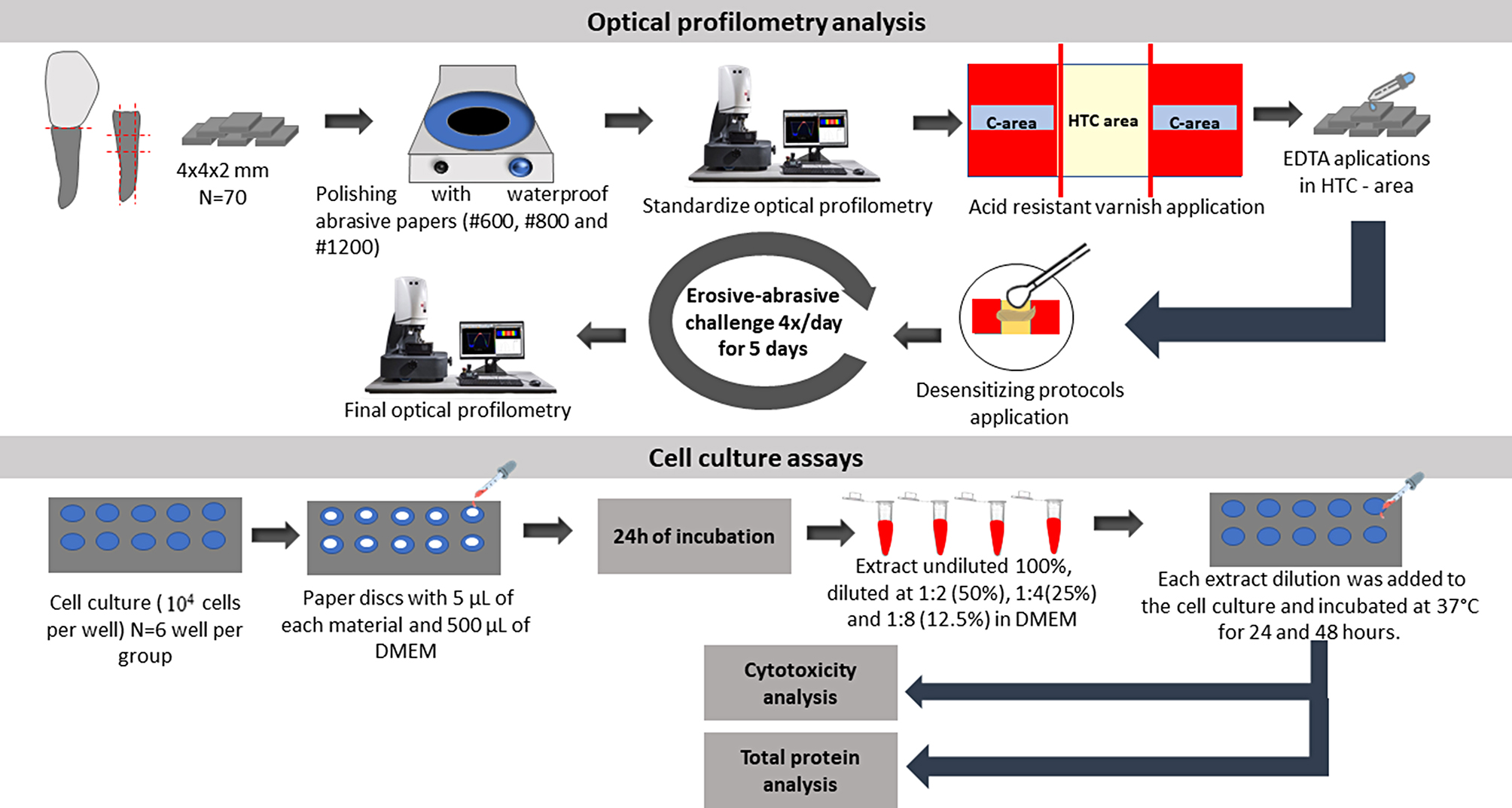
Study design illustration. Dentin wear surface were assessed using an optical profilometer. Control (C), DOT – dentin with opened tubules (EDTA immersion), treated (with desensitizing agents), challenged (erosive-abrasive cycles)

Regarding sample size calculation, a pilot study was conducted to determine the number of specimens per group for dentin wear analysis. Sample size was calculated using three specimens from pilot study performed by an experienced operator. ANOVA for sample size test was used adopting α = 0.05 and a power of 0.80, with an expected difference between means of 6.8 (SigmaPlot 13 software Systat Software Inc., London, UK). A sample size of 10 specimens per group was found.

### Dentin wear analysis

#### Dentin sample preparation

Seventy freshly extracted bovine incisors were collected and teeth with caries, cracks, or gross irregularities of dentin structure were excluded from the study.

From each tooth, a root dentin block was obtained with dimensions of 4 × 4 × 2 mm (*n* = 70). The dentin blocks were polished with waterproof abrasive papers (#600, #800 e #1200 grit), in a polishing machine (AutoMet 250 PRO, Buehler, IL, USA), under running water. The dimensions were checked with a digital micrometer (Mitutoyo America, Dawn, IL, USA). The specimens were ultrasonically cleaned for 5 min between each abrasive paper (Cristófoli, Campo Mourão, PR, Brazil). In order to standardize the specimens, dentin blocks were analyzed with optical profilometry (Proscan 2100, Scantron Ltd, Venture Way, Taunton, UK) to discard specimens with curvature values higher than 0.3 μm [24]. Then, the polished surfaces were protected using an acid-resistant varnish (Colorama, São Paulo, SP, Brazil), leaving a central area of 4 × 1 mm exposed to receive the treatments and two lateral areas of 4 × 1.5 mm as control surfaces [24]. The smear layer was removed in the central area using 0.5 M Trisodium ethylenediaminetetraacetic acid (EDTA) in concentration of 17.5% and pH 7.3 solution for 5 min, to open dentin tubules [10]. The following areas were created: one central area - DOT – dentin with opened tubules (EDTA immersion), treated one of the desensitizing agents, challenged (erosive-abrasive cycles); and two lateral areas - C – control (no treatment) [27]. Only areas C received the acid-resistant varnish.

#### Experimental groups and modes of application

The samples of dentin were randomly separated in seven experimental groups (*n* = 10): placebo varnish (PLA), fluoride varnish (FLU); nanoparticulate sodium trimetaphosphate varnish (TMP); universal adhesive (SBU); surface pre-reacted glass-ionomer filler-containing varnish (SPRG); bioactive ceramic solution (BIOS) and protein from enamel solution (AMTN).

All HTC-area received all desensitizers under clean and dry surface with a disposable applicator (KG Sorensen, Cotia, SP, Brazil). In the samples from the PLA, FLU and TMP groups a thin layer was passively applied for 5 s, remaining stable for 10 min. In SBU samples, a thin layer was actively applied for 20 s and photocured using light intensity of 1200 mW/cm^2^ (Radii, SDI, Victoria, Australia) for 10 s, without previous acid etching. One drop of varnish was actively mixed with base and applied for 3 s for SPRG group, forming a thin layer. The same light device was used during 10 s. The uncured layer was removed from surface with the cotton pellet. In the BIOS group a thin layer of bioactive ceramic solution was applied for 5 s, remaining stable for 10 min. In AMTN samples, 5 µL of solution were applied for 10 s, remaining for 10 min.

A single experienced researcher was trained and calibrated to perform the specimens in a previous research and also in the pilot study procedures. This researcher performed all applications only once and the specimens were immediately stored in artificial saliva (1.649 mmol/L CaCl_2_ H_2_O, 5.715 mmol/L KH_2_PO_4_, 8.627 mmol/L KCl, 2.950 mmol/L NaCl, l.92 mmol/L Tris buffer, pH adjusted to 7 with HCl) for 6 h at 37 °C [10].

#### Erosive-abrasive challenge

During the experimental period, specimens were subjected to a 5-day erosive-abrasive challenge. Erosive cycles were performed four times daily, and abrasive challenges were applied after the first and last erosive cycles. The samples were eroded by immersion in 2 ml/block of citric acid (pH = 3.2) for 2 min under an orbital shaking table (Tecnal TE – 420, Piracicaba, SP, Brazil) [10] with 1 h immersion in artificial saliva (1.649 mmol/L CaCl_2_ H_2_O, 5.715 mmol/L KH_2_ PO_4_, 8.627 mmol/L KCl, 2.950 mmol/L NaCl g/l.92 mmol/L Tris buffer, pH adjusted to 7 with HCl) between the cycles [10].

Abrasive challenge was performed by brushing the specimens for 15 s using an automated machine (MSET, Elquip, São Carlos, SP, Brazil), at 150 strokes/min during 2 min and axial load of 150 g [10]. For all groups, brushing was performed with a slurry made from Colgate Total 12 (Colgate-Palmolive, São Paulo, SP, Brazil) dentifrice and artificial saliva (1:3 w/w) [10]. At the end of experiment period, the samples were stored under 100% humidity until analysis.

#### Final wear analysis

After abrasive cycles, the acid-resistant varnish was mechanically removed with a flat and thin #15c scalpel blade (Solidor, São Paulo, SP, Brazil). The dentin wear was determined with optical profilometer programmed to scan a central area of the specimen 2 mm long (x-axis) by 1 mm wide (y-axis), being 1 mm reading from the HTC area and 0.5 mm from the C areas on each side (Fig. 1). The equipment was set to go 200 steps of 0.01 mm on the x-axis, and 20 steps of 0.05 mm on the y-axis, using a specific software (Proscan Application software v. 2.0.17, Scantron, Venture Way, Tauton, United Kingdom). The dentin wear was calculated based on subtracting the mean height of the test area (HTC) from the mean height of the two reference areas (C). For this analysis, a 3-point height tool was applied. To avoid collagen shrinkage of dentin, specimens were scanned in a moistened condition. The result was expressed in micrometers [27].

### Biological properties

#### Growth cell conditions

To evaluate the cell response to experimental materials, immortalized cells of the gingival fibroblast cells line (NHI/3T3 – ATCC CRL-1658) and odontoblast-like cells line (mouse dental papila cells - MDPC-23) were used. These cells were cultured (Costar Corp., Cambridge, Massachusetts, USA) in Dulbecco´s Modified Eagle´s Medium (DMEM, SIGMA Chemical Co., St. Louis, Missouri, USA) containing 10% fetal bovine serum (SFB, Cultilab, Campinas, SP, Brazil), 100 UI/mL e 100 µg/mL, penicillin and streptomycin, respectively (GIBCO, Grand Island, Nova York, USA). The cells were incubated at 37 °C in a humidified atmosphere of 5% of CO_2_ and 95% air [28]. Culture media were renewed every 2 days until cells reach 80% confluence. Then, cells were grown in 75 cm^2^ flasks up to reach 80% confluence and then they were detached using 25% trypsin-EDTA (GIBCO, Grand Island, Nova York, USA). After being recovered, washed and re-suspended in a 15mL tube, 100 µl of cells were stained with 900 µl of 0.4% Trypan-blue staining in PBS solution to determine total cells count/mL using an automatic cell counter (TC20 Automated Cell Counter, Bio-Rad, Santo Amaro, São Paulo, SP, Brazil). Based on the total cell count, the website Cell Plating calculator (https://www.axionbiosystems.com/cell-plating-calculator) was used to determine the volume of media to dilute the initial cell suspension to achieve 1 × 10^4^ cells/well for being seeded in 96-well plates (Kasvi, São José dos Pinhais, PR, Brazil) [28, 29].

#### Experimental groups and modes of application

Paper discs with 6 mm diameter were sterilized and impregnated with 5 µL of each material (Table 1) such as described previously [22].

Subsequently, the discs were inserted in microtubes containing 500 µL of DMEM and kept at 37 °C for 24 h according to ISO10993-12-2021 (https://www.iso.org/standard/53468.html). After this period, the extract (100%) of each material as well as dilutions at 1:2 (50%), 1:4(25%) and 1:8 (12.5%) in DMEM prepared to be applied to cell cultures [30]. A control group with DMEM with no extracts was included in the study. The culture medium in each well was subsequently removed and 100ul of each extract was added to the cell cultures and incubated at 37 °C for 24 h [12].

**Table 1:** Characteristics and mode of application of in-office desensitizing materials used in this study

Materials	Main ingredients	Manufacturer	Batch #	Application
Placebo varnish (PLA)	Artificial resin, solvent, essence, saccharine, and deionized water.	SS White Dental Products	-	A thin layer was passively applied for 5 s under the clean and dry surface with a disposable applicator, remaining stable for 10 min.
Fluoride varnish (Duraphat – FLU)	5% NaF (22.600 ppm), colophony; solvent, shellac; mastic; saccharine and others.	Colgate-Palmolive Company	022001	
TMPnano varnish (TMP)	NaF 5%+5% TMPnano (22.7 nm); artificial resin, solvent, essence, saccharine, and deionized water.	SS White Dental Products and Sigma-Aldrich	-	
Universal Single Bond (SBU)	BISGMA; HEMA; UDMA; DPIHFP, 10-MDP; solvent; water; silane; and others.	3 M ESPE	1,833,100,782	A thin layer was actively applied for 20 s on the clean and dry surface with a disposable applicator and light-cured for 10 s. No previous acid etching was performed.
Barrier Coat (S-PRG filler varnish – SPRG)	S-PRG filler (3.0 μm): TEGDMA; Bis-MPEPP; fluorine boron aluminosilicate; MAA; phosphonic acid; and others.	Shofu INC.	121,901	A thin layer of one drop active mixed with base in the base container was applied for 3 s on the clean and dry surface with specific applicator and light-cured for 10 s. The uncured layer was removed from surface with a water-moistened cotton pellet.
Biosilicate solution (BIOS)	The solution was composed of Biosilicate powder (P_2_; O_5_-Na_2_; O-CaO-SiO_2_ 1–10 μm) and distilled water (1:10 ratio) and for simulation of the professional-use products, the particles were mixed immediately before application	Laboratory of Vitreous Materials at the Federal University of São Carlos	-	A thin layer was applied for 5 s on the clean and dry surface with a disposable applicator, remaining stable for 10 min.
Amelotin solution (AMTN)	Protein derived from dental enamel. The solution was prepared with 100 µL of pure water added to 500 µg of AMTN powder. The result was 200 µL of solution (5 µg/µL concentration).	Institute of Biomedical Engineering, University of Toronto	-	5 µL of solution were applied for 10 s on the clean and dry surface with a disposable applicator, remaining stable until no visible liquid left.

Abbreviations TMPnano (nanoparticulate sodium trimetaphosphate) TEGDMA (triethylene glycol dimethacrylate); BISGMA (diglycidildimethacrylate A); HEMA (Hydroxyethylmethacrylate); UDMA (1,3 glycol dimethacrylate) DPIHFP (Diphenyliodonium hexafluorophosphate); 10-MDP (10-decanediol phosphate methacrylate); Bis-MPEPP (bisphenol A polyethoxy methacrylate); MAA (methacrylic acid

### Cytotoxicity tests

For cytotoxicity assessment, L3T3 and MDPC-23 cells were exposed to different extracts (extract and diluted from 1:2 to 1:8 in DMEM). After 24 h of cell exposure, 125µL of culture medium containing resazurin solution (at 70µM) was added to each well. Viable cells reduced resazurin (blue color) to resorufin (pink color), and the production of resorufin was proportional to the metabolic activity of the viable cells. After 4 h, 100 µL of the resazurin-culture medium solution was transferred to a 96-well plate for reading in a spectrophotometer (Spectra Max 190; Molecular Devices, Sunnyvale, California, USA) at 570 and 600 nm [31]. Cell viability was calculated from the control group without treatment (DMEM) which was considered as 100%. According to the other study, the cell viability will be discussed considering the parameters cited as follow: non-cytotoxic (more than 90% cell viability), slightly cytotoxic (60–90% cell viability), moderately cytotoxic (30–59% cell viability) and severely cytotoxic (less than 30% cell viability) [23].

### Total protein production (TP)

To determine the total protein (TP) production, L3T3 and MDPC-23 cells were also seemed in a 96-well plate and exposed to extracts (extract and diluted in DMEM) of each desensitizer agent for 24 h, as described previously. After these periods, the culture medium was removed and 150 µL of 0.1% sodium lauryl sulfate in deionized water (Sigma / Aldrich Corp., St. Louis, MO, USA) was added to each well and kept for 40 min at room temperature to produce cell lysis. Then, 100µL of this solution was pipetted into a 96-well plate and 50 µL of Lowry Reagent Solution (Sigma / Aldrich Corp., St. Louis MO, USA) was inserted to each well and incubated for 20 min at room temperature. Afterwards, 25 µL of Folin-Ciocalteu Phenol Reagent Solution (Sigma / Aldrich Corp., St. Louis MO, USA) was added to each well and kept for 30 min. The absorbance values of the wells were determined at a wavelength of 655 nm in a spectrophotometer. TP production was calculated from a standard curve using pre-determined bovine serum albumin (BSA) concentrations [28].

### Statistical analysis

Normal distribution and homoscedasticity of the data was checked using Shapiro–Wilk and Brown-Forsythe tests, respectively. Data from dentin wear did not present a normal distribution; thus, comparisons were performed using Kruskal-Wallis and Dunn’s tests. The cytotoxicity data were expressed in percentage of cell viability in relation to the control with no treatment (DMEM medium − 100% of cell growth). Data from TP were expressed in ug/mL. Cytotoxicity and TP were evaluated by ANOVA One-Way and Tukey tests. Spearman correlations were also conducted between cytotoxicity and TP data. The software used for statistical analysis was Jamovi version 2.2.5 (Sydney, Australia), with a significance level of 5%.

## Results

### Dentin wear

**Fig. 2:**
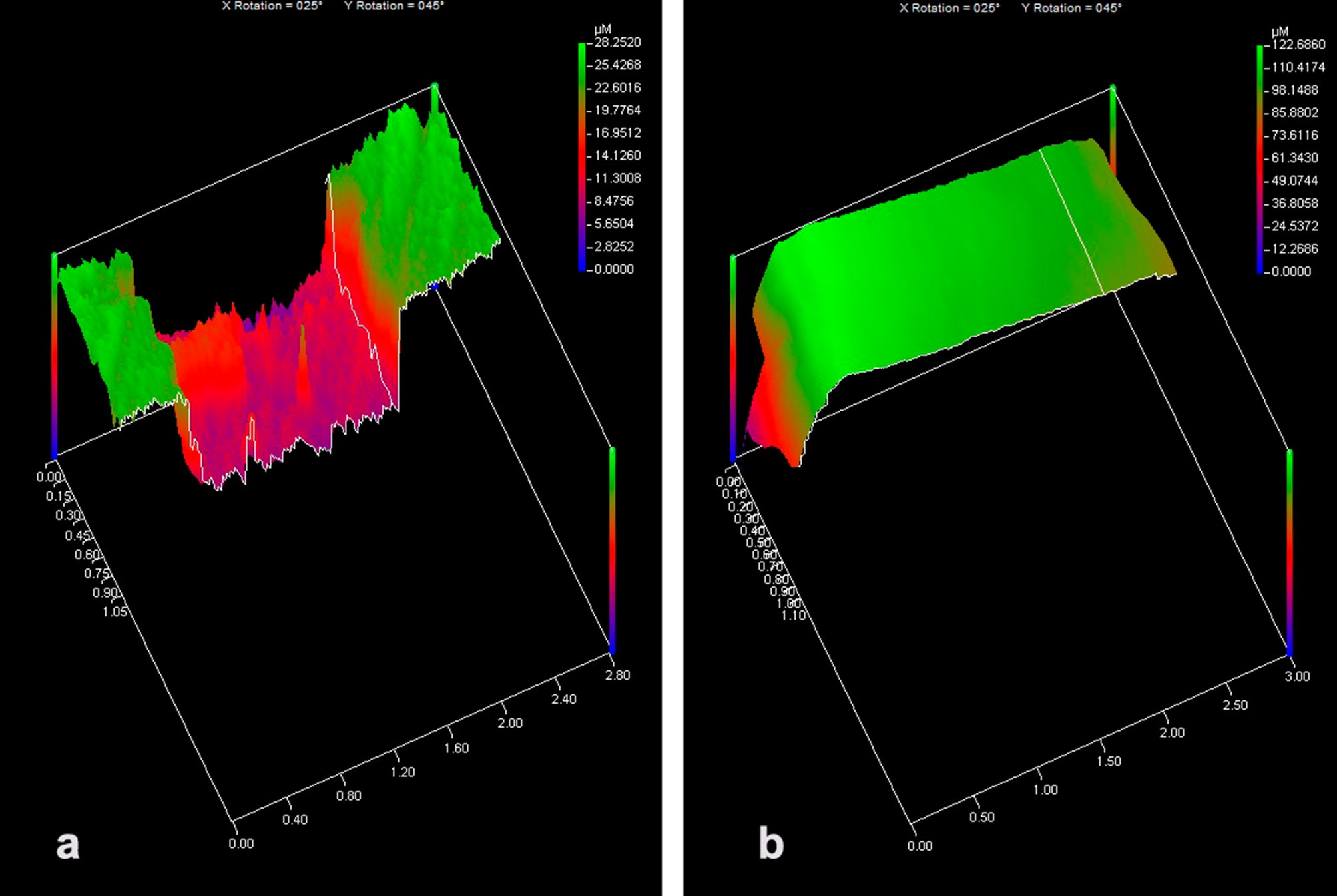
Representative images of the 3-D plot of surface loss from PLA group (a) and surface preservation from SBU group (b) after erosive-abrasive challenge

The results for dentin wear can be observed in Table 2 and representative images in Fig. 2. No wear was presented only for SBU, being statistically different to the other materials (*p* < 0.001). The lowest wear was obtained for AMTN, being statistically similar to TMP (*p* > 0.05).

**Table 2:** Data referring to optical profilometry in (Curvature in µm) of the different *in-office* desensitizers after the erosive-abrasive challenge

Materials	Mean	±SD	Median (25%/75%)	Comparison
PLA	-9.05	1.71	-9.42 (-10.25/-8.9)	D
FLU	-5.11	1.30	-4.66 (-6.41/-4.44)	C
TMP	-3.07	1.15	-3.19 (-3.82/-2.75)	BC
SBU	34.18	20.68	25.89 (22.46/51.96)	A
SPRG	-3.68	0.97	-4.05 (-4.28/-2.94)	C
BIOS	-3.87	1.19	-4.14 (-4.74/-3.02)	C
AMTN	-1.41	1.14	-0.75 (-2.15/-0.72)	B

Different letters indicate a statistically significant difference (*p* ≤ 0.05). PLA: Placebo varnish; FLU (Fluoride varnish); TMP (TMPnano varnish); SBU (Universal Single Bond); SPRG (Barrier Coat); BIOS (Biosilicate solution); AMTN (Amelotin solution). Kruskal-Wallis and Dunn’s tests were performed

### Cytotoxicity analysis

**Fig. 3:**
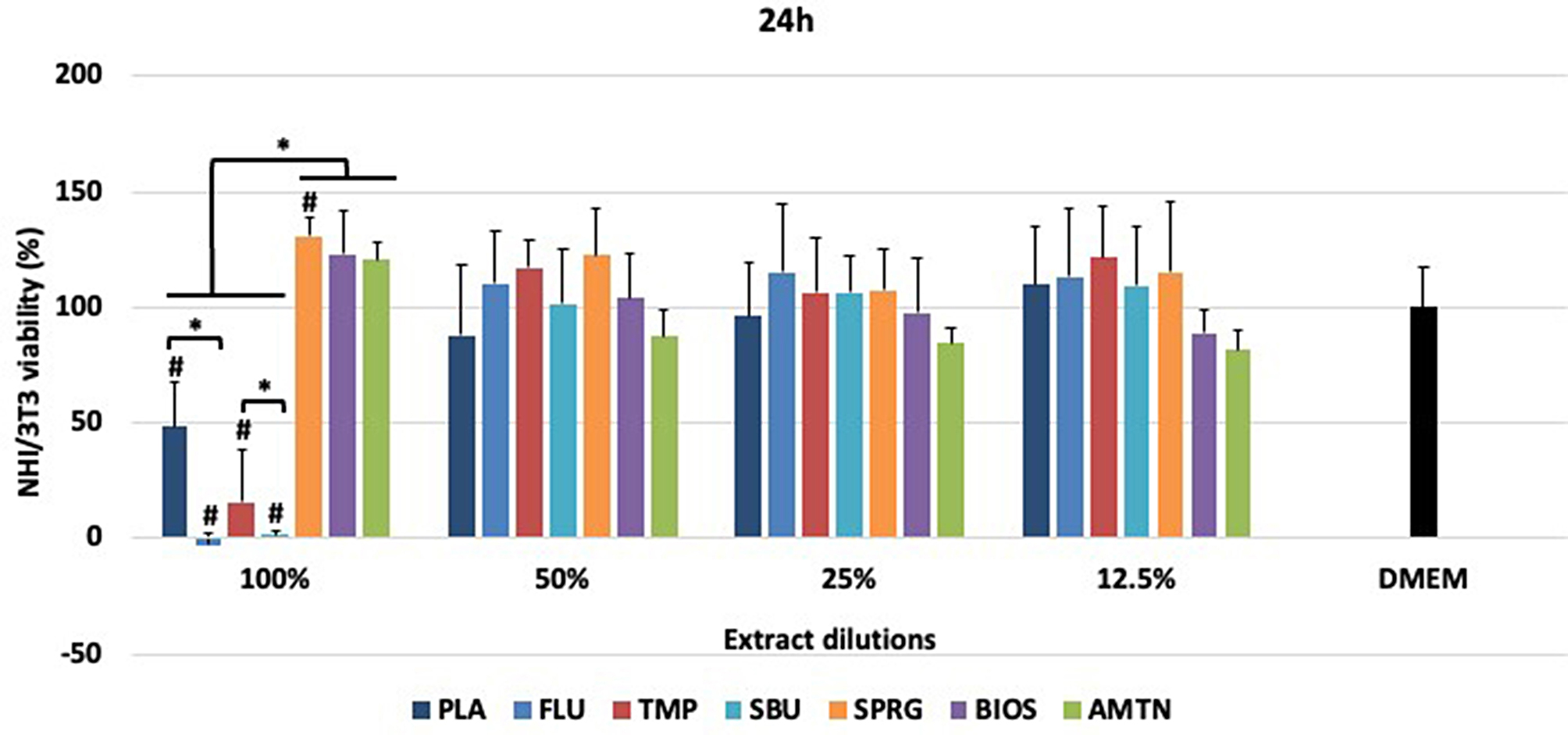
Percentage of NHI/3T3 cell viability (means ± standard deviations deviation) after treatments with different materials.*Statistical difference among the groups of materials, considering each dilution separately, according to One-Way ANOVA and Tukey test, considering *p* < 0.05. # Statistical difference between DMEM and each material, at all dilutions, according to One-Way ANOVA and Tukey test, considering *p* < 0.05.100% - extract, 50% diluted, 25% diluted, 12.5% diluted in DMEM. Placebo varnish (PLA), fluoride varnish (FLU); nanoparticulate sodium trimetaphosphate varnish (TMP); universal adhesive (SBU); surface pre-reacted glass-ionomer filler-containing varnish (SPRG); bioactive ceramic solution (BIOS) and protein from enamel solution (AMTN)

**Fig. 4:**
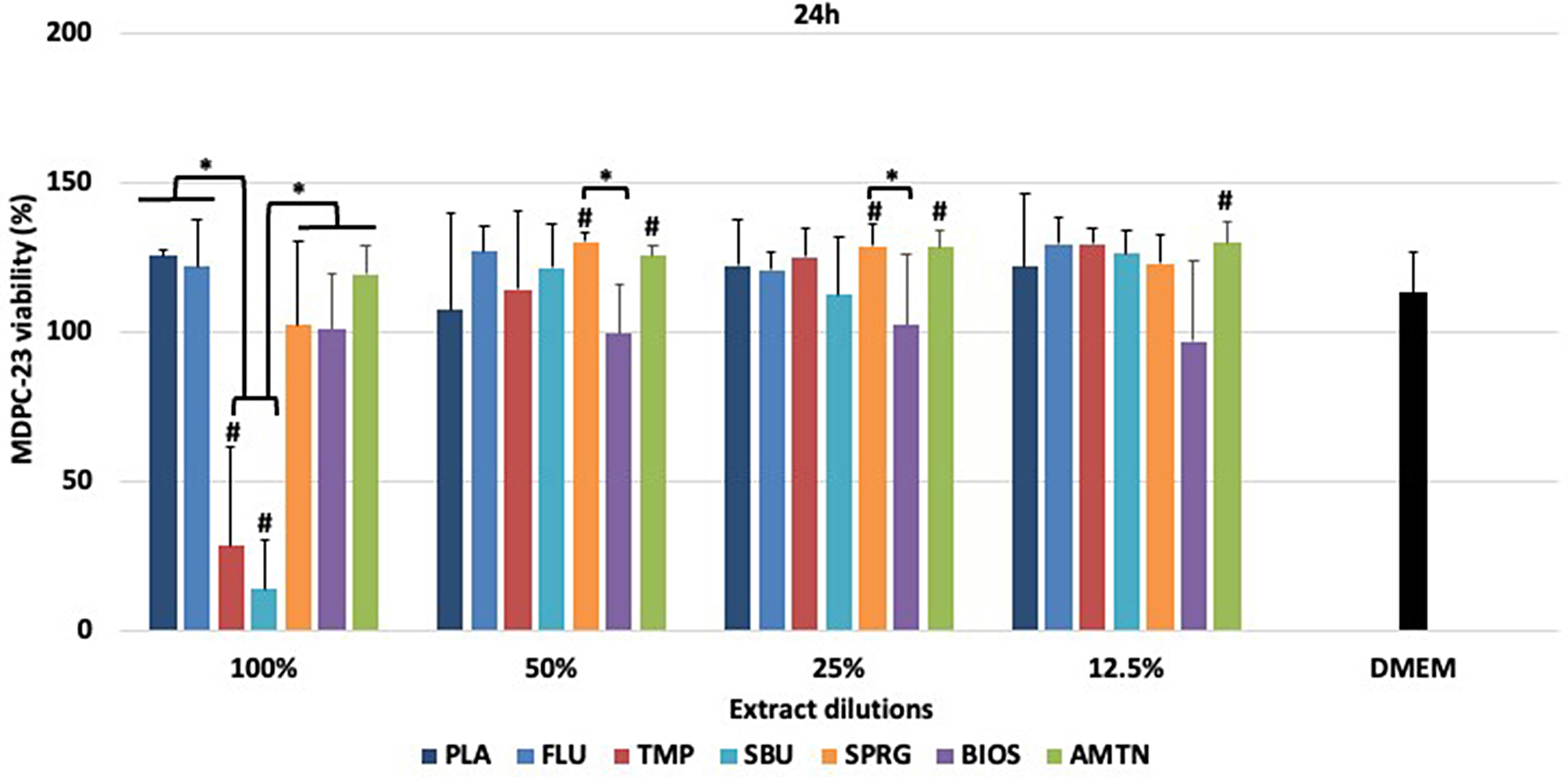
Percentage of MDPC-23 cell viability (means ± standard deviations) after treatments with different materials.*Statistical difference among the groups of materials, considering each dilution separately, according to One-Way ANOVA and Tukey test, considering *p* < 0.05. # Statistical difference between DMEM and each material, at all dilutions, according to One-Way ANOVA and Tukey test, considering *p* < 0.05. 100% - extract, 50% diluted, 25% diluted, 12.5% diluted in DMEM. Placebo varnish (PLA), fluoride varnish (FLU); nanoparticulate sodium trimetaphosphate varnish (TMP); universal adhesive (SBU); surface pre-reacted glass-ionomer filler-containing varnish (SPRG); bioactive ceramic solution (BIOS) and protein from enamel solution (AMTN)

Data from cytotoxicity assays after 24 h of materials treatments are presented in Figs. 3 and 4 for NIH/3T3 and MDPC-23 cells, respectively.

Low cell viability was observed for FLU, TMP and SBU (*p* < 0.05) with no differences between TMP and PLA (*p* > 0.05), when NHI/3T3 were exposed to material’s extracts with no dilution (100%) (Fig. 3). No differences were found among materials when diluted extracts were evaluated (*p* > 0.05). FLU, TMP, SBU and SPRG statistically differed from the control (DMEM). SPRG, BIOS and AMTN were cytocompatible at all dilutions tested.

TMP and SBU desensitizers also caused higher toxicity to MDCP-23 compared to the other materials and control (DMEM) when cells were exposed to extract (100%) (*p* < 0.001) (Fig. 4). Considering 50% and 25% dilution, BIOS demonstrated significantly less cell viability than SPRG; however, it did not differ from the other materials. There was no difference between materials when they were tested at 12.5% dilution (*p* > 0.05). Cell activity statistically increased after exposure to SPRG at 50 and 25% dilution and AMTN from 50 to 12.5% dilution compared to control (DMEM).

Figures S1 and S2 showed the categorization of materials according to their effect on NHI/3T3 and MDPC-23 cell viability (in scores). Considering NHI/3T3 cells, the materials were ranked from non-cytotoxic or slightly cytotoxic (SPRG, BIOS and AMTN) to moderately or severely cytotoxic (PLA, FLU, TMP and SBU) at 100% extract. However, after dilution, all the materials were classified as non-cytotoxic or slightly cytotoxic. For MDPC-23, only TMP and SBU were ranked as moderately or severely cytotoxic at 100% extract. After dilution, these materials were classified as non-cytotoxic or slightly cytotoxic. The other materials (PLA, FLU, SPRG, BIOS and AMTN) were ranked as non-cytotoxic or slightly cytotoxic at all concentrations tested.

### TP analysis

**Fig. 5:**
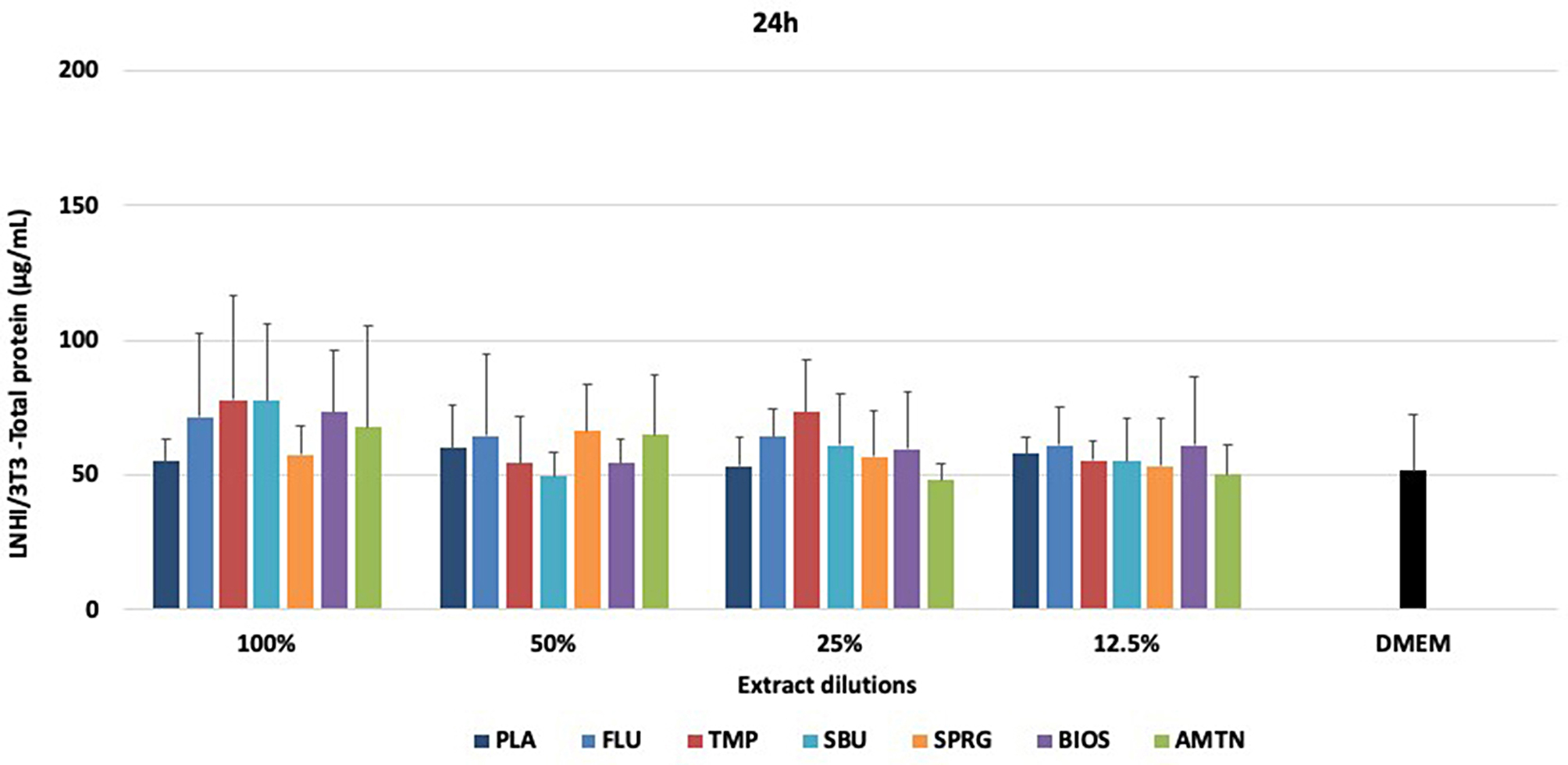
Total protein concentrations (µg/mL; means ± standard deviations ) obtained by L3T3 after treatments with different materials. No statistical difference was observed among the groups and when they were compared to DMEM. *Statistical difference among the groups of materials, considering each dilution separately, according to One-Way ANOVA and Tukey test, considering *p* < 0.05. # Statistical difference between DMEM and each material, at all dilutions, according to One-Way ANOVA and Tukey test, considering *p* < 0.05.100% - extract, 50% diluted, 25% diluted, 12.5% diluted in DMEM. Placebo varnish (PLA), fluoride varnish (FLU); nanoparticulate sodium trimetaphosphate varnish (TMP); universal adhesive (SBU); surface pre-reacted glass-ionomer filler-containing varnish (SPRG); bioactive ceramic solution (BIOS) and protein from enamel solution (AMTN)

**Fig. 6:**
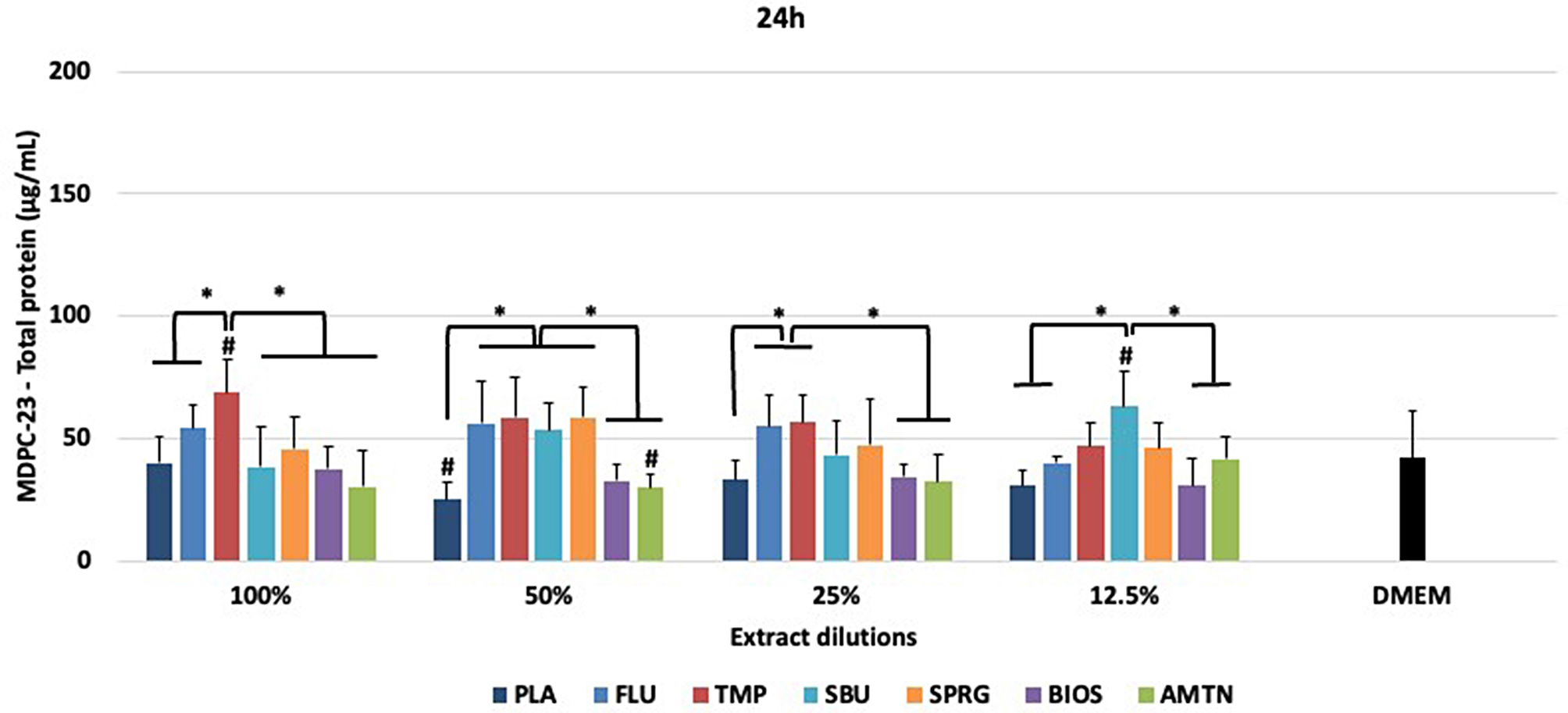
Total protein concentrations (µg/mL) obtained by MDPC-23 after treatments with different materials. *Statistical difference among the groups of materials, considering each dilution separately, according to One-Way ANOVA and Tukey test, considering *p* < 0.05. # Statistical difference between DMEM and groups of materials, at all dilutions, according to One-Way ANOVA and Tukey test, considering *p* < 0.05. 100% - extract, 50% diluted, 25% diluted, 12.5% diluted in DMEM. Placebo varnish (PLA), fluoride varnish (FLU); nanoparticulate sodium trimetaphosphate varnish (TMP); universal adhesive (SBU); surface pre-reacted glass-ionomer filler-containing varnish (SPRG); bioactive ceramic solution (BIOS) and protein from enamel solution (AMTN).

Figures 5 and 6 show the total protein concentrations determined for NHI/3T3 and MDPC-23 cells, respectively, after 24 h of materials treatments.

Considering NHI/3T3 cells (Fig. 5), there were no statistical differences among the materials tested at different concentrations and when the materials were compared to DMEM (*p* > 0.05). In relation to MDPC-23 cells (Fig. 6), TMP extract (100%) induced higher production of TP than other groups. FLU, TMP, SBU and SPRG groups at 50% also showed the highest TP concentrations compared to the other groups (*p* < 0 0.001). At 25% dilution, the highest protein production was observed for FLU and TMP groups (*p* = 0.005), being statistically similar to SBU and SPRG (*p* > 0.05). At 12.5% dilution, SBU showed highest TP concentration (*p* = 0.004) with no statistical difference compared to TMP and SPRG (*p* > 0.05). TMP at 100%, PLA and AMTN at 50% and SBU at 12.5% dilution statistically differed from the control (DMEM).

Correlations between data from cell viability and total protein analysis are presented on Tables S1 and S2 for NHI/3T3 cells and MPCD-23, respectively, considering each extract of material separately. For NHI/3T3 cells, positive correlations between those data were observed after BIOS treatments at all concentrations (from 100 to 12.5%), after SPRG treatment at 100% extract and 25% dilution and negative correlation for TMP at 25% dilution. Considering MDPC-23 analysis, positive correlation between cell viability and TP data were observed for SPRG at 100% and negative correlation were noted for BIOS 12.5%.

## Discussion

This study aimed to evaluate the dentin wear protection and biological properties of different in-office desensitizing agents, in special a protein from enamel named amelotin. The findings support the hypothesis that this enamel protein demonstrated low dentin wear, presenting biocompatibility, being a potential material for use to treat dentin with opened tubules.

Several materials have been studied in the treatment of erosive tooth wear, which should be able to reduce dentin hypersensitivity, as well as protect dentin from erosive/abrasive challenges [5, 6]. Some analysis has been proposed to analyze the in vitro performance of desensitizers [32], among them 3D optical measurements have been suggested how a more accurate assessment than other techniques for examining surface specimen alteration [32].

As expected, PLA showed the worst results in preventing dentin wear if compared to the other groups. In contrast, no wear was found for SBU maintaining the material over the surface, rejecting the first null hypothesis. The literature has shown that varnishes containing different sources of fluoride can reduce dentin wear compared to PLA [15, 24, 33]. The FLU showed no differences compared to TMP, BIOS and SPRG. The FLU varnish may promote the precipitation of a layer similar to calcium fluoride (CaF_2_) [11, 24]. CaF_2_ acts as a physical barrier which can prevent acid action on dentin surface [11]. However, the effectiveness of fluoride varnish was compromised after erosive/abrasive challenge, such as demonstrated by the results found in this study and in another previous study [11]. The TMP was used to improve the effectiveness of fluoride varnish because it is a cyclophosphate which produces a negative surface polarity, increasing the deposition of CaF_2_ [14, 15]. The similarity between FLU and TMP can be explained because the effect of fluoride on dentin does not depend only on the deposition of large amounts of fluoride. When the collagen matrix is removed, hydrogen can easily penetrate in the porous dentin, causing severe mineral loss even in the presence of fluoride [34]. In contrast, TMP associated with fluoride varnish promoted less dentin wear after erosive/abrasive challenge compared to 5 and 2.5% NaF varnish using contact profilometry [15].

Concerning photoactivated products, SBU presented a superior result compared to SPRG, with formation of a positive curvature, which indicates permanence of material on the dentin surface even after erosive/abrasive challenge. This can be explained because acidic monomers of self-etch adhesives promotes the simultaneous dissolution of smear layer and creates a hybrid layer without exposing the collagen fibrils, reducing the risk of collapse of the collagen network, and sealing the dentin tubules [16, 35]. In another study of our research group, only the SBU under the same challenge, maintaining hydraulic conductance [10]. In a previous study, SPRG material demonstrated the ability to protect the dentin surface from demineralization after immersion in acid medium [36]; however, when the erosion is associated to abrasion, this material did not resist on the dentin surface such as observed in the present study. According to the manufacturer, this material was developed to be applied on both enamel and dentin, and due to the phosphonic acid monomers, its retention to the tooth surface occurs by a chemical interaction with hydroxyapatite crystals [37]. In this study, dentin specimens were gently dried with an absorbent paper, which better represents one of the steps of the procedures used in dental clinical for treatment of dentin with opened tubules. SPRG contains TEGDMA with other monomers that increase the product’s viscosity [37]. It is also worth mentioning that TEGDMA is an ester-based hydrophilic monomer, susceptible to hydrolytic degradation [38]. This hydrolysis results in disruption of the inter-molecular bonds, plasticizing the polymer chain over time which could lead to leaching of monomers within the dentinal tubules [38]. In another study, show high permeability, since the dentin surface became rapidly wet when specimens were kept in a machine that simulated the dental pulp pressure [37]. SPRG was not efficient in protect dentin surface after erosive/abrasive challenge probably due the detached polymeric layer on the surface that still allowed water flow underneath.

In relation to experimental materials, BIOS showed a higher dentin wear than AMTN. AMTN acts by biomineralization and dentogingival attachment, promoting calcium phosphate precipitation, formation of dose-dependent hydroxyapatite and collagen matrix [19, 20] which appeared able to protect the surface from wear. Regarding the BIOS, this material was capable of controlling the progression of erosion lesion when submitted to erosive challenge [32]. Comparing BIOS and AMTN in other study, no differences were found between them when mean length of occluded dentinal tubules were analyzed [10]. However, when dentin permeability and scanning electron microscopy analysis were performed, AMTN showed better results than BIOS [10].

It is important to highlight that desensitizers agents should also present biocompatibility with adjacent tissues in order to be safe and effective [23]. The evaluation of the cytotoxicity of these materials, improves the understanding of their mechanism of action, considering that two of these materials are experimental. In this context, cells used in this study are related to the cervical area where these materials are applied, specifically gingival fibroblasts (due to the presence of gingival tissue in these regions) and odontoblast-like cells (due to the presence of odontoblastic inside the dentinal tubules) [30]. Additionally, the present study used dilution of extracts to simulate the interposition of the dentinal barrier, since it is known that the desensitizers did not reach cells in original concentration [22, 30].

Nowadays, there is a degree of opposition to fluoridation due to the risk of possible toxicity [39]. In this study, FLU in extract (100%) was ranked as severely cytotoxic to NHI/3T3 cells, corroborating with previous studies that demonstrate that undiluted extracts of fluoride varnishes are toxic to fibroblast cells [30, 40]. A previous study suggests that other components present in fluoride varnishes may also influence their biological responses, which would justify the results of PLA [30]. TMP (at 100% extract) were classified as moderately or severally cytotoxic to both NHI/3T3 and MDPC-23 cells. A recent study demonstrated that TMP can exert a physicochemical effect in inducing the formation of hydroxyapatite crystals, however, it interferes negatively in the gene expression of odontoblastic cells, which may justify the reduction in their metabolism [41]. However, these materials can be considered safe after dilution because they were non-cytotoxic for both cells from the 50% dilution [30, 40].

Considering photocured agents, SBU were classified as severely cytotoxic to both cells when they were treated with undiluted extract, corroborating with the results observed in the literature [22, 42, 43]. Cells cultivated with self-etching adhesives tend to show an increase in apoptotic activity, which can be explained by the high acidification of the medium due to the presence of monomers methacryloyloxydecyl dihydrogen phosphate (MDP) [42, 43]. A previous study demonstrated low values of cell viability for SBU, with means around of 2%, corroborating with this study which has an average of 1.4% [43]. A significant reduction in cell metabolism after 24 h of contact with the extract obtained from impregnated filter paper discs also was found in a study that evaluated the cytotoxicity of experimental adhesive with different degrees of hydrophilicity in odontoblast cell culture [44]. The use of universal adhesives had not been recommended for deep dentin due to their high toxic potential to pulp cells [45]. However, other study demonstrated that improvements in universal adhesive system formulations and their mechanisms of action are not accompanied by increased toxicity compared with those in other systems, warranting commitment to the use of these materials on dentin-pulp complex [26]. In relation to SPRG, it is difficult to compare the results of present study with the literature because only the S-PRG filler elute have been studied in relation to the cytotoxicity [46, 47]. These studies suggested that this material could be applied in dental practice because the safety of SPRG eluate was identified for fibroblast and odontoblast-like cells [45, 47].

AMTN and BIOS groups showed a non-cytotoxic or slightly cytotoxic effect on both cells analyzed. Probably, the AMTN results occurred due to the composition of this material is based on the protein expressed in the maturation of enamel [19]. Regarding the BIOS, previous study also demonstrated that this material has no cytotoxic effects [25]. It is important to highlight that the experimental solutions used in the present study did not present some components, such as monomers and solvents, which can produce an apoptotic cellular response [22, 30, 40, 42, 43].

TP analysis aims to investigate the functionality of the materials tested [18, 28]. The release of TP in the cell culture supernatant can provide information on cell physiology and also on its productivity, being a biocompatibility marker complementing the results that are obtained by cytotoxicity analysis [48]. In this study, no statistical difference was observed among the groups of materials for TP produced by NHI/3T3 cells, independent on the concentration, correlations analysis observed a positive association between cell viability and TP production after SPRG and BIO treatments. These results could indicate that both materials stimulate protein expression and cell activity increasing the repair mechanisms and pulp healing after removal of diseased dental tissues and clinical restoration of the tooth. It is stated that dentin barrier formation only occurs when pulp inflammation and infection are controlled promoting the reestablishment of pulp health [49]. For MDPC-23, TMP and FLU groups induced higher protein production, both at 50 and 25% dilution, being significantly different from the other desensitizing agents, suggesting higher metabolic activity [18]. However, no correlations between cell viability and TP production were observed for those materials.

In the current study, some aspects might be considered as limiting factors, such as the use of bovine teeth, the temperature of the oral cavity, the presence of occlusal forces, the clinical buffering capacity of saliva, and the presence of proteins of dentin and saliva; then, the mechanical, enzymatic and microbiological effects could not be expected [24]. Additionally, the extracts used in this study were applied directly on the cells, without the dentin protection. It’s worth considering that the oral epithelium at the gingival margin has a high rate of renewal and dentinal fluid is present inside the dentinal tubules. Furthermore, care must be taken when extrapolating our results to the clinical setting, where several local factors may influence the results.

Additional tests on the dentin structure’s surface, including x-ray diffraction, Fourier Transform Infrared Spectroscopy, and Raman, would enhance the validity of the conclusions and establish a more robust foundation for this study’s findings. A long-term clinical trial is also necessary to define dentin hypersensitivity reduction and dentin protection of these materials. Besides, different bio-active polymers have been launched in the market, as alternative to fluoride mediated desensitization, being possible the at home (patient-applied) therapy [50].

## Conclusions

Considering the limitations of this study, universal adhesive system may protect the wear of dentin with opened tubules after erosive-abrasive challenge. However, this adhesive system and fluoride varnishes may be cytotoxic in undiluted extract, mainly for fibroblast cell. The enamel protein may be a future alternative to treat dentin with opened tubules because it may cause low wear under erosive-abrasive challenge with low cytotoxic effects.

### Electronic supplementary material

Below is the link to the electronic supplementary material.

Supplementary Material 1

## Data Availability

The datasets analyzed during the current study are available from the corresponding author on reasonable request.

## Abbreviations

AMTN: Amelotin
ANOVA: Analysis of variance
BIOS: Biosilicate
C: Control
DMEM: Dulbecco´s Modified Eagle´s Medium
EDTA: Ethylenediaminetetraacetic acid
FLU: Fluoride varnish
HTC: Hypersensitive (EDTA immersion), treated (with desensitizing agents), challenged (erosive-abrasive cycles)
MDP: Methacryloyloxydecyl dihydrogen phosphate
MDPC-23: Odontoblast-like cell
NCLC: Non-carious cervical lesions
L3T3: ATCC CRL-1658 = Fibroblast-like cell
PLA: Placebo varnish
SBU: Universal adhesive
SPRG: S-PRG varnish
TEGDMA: Triethylene glycol dimethacrylate
TMP: Sodium trimetaphosphate
TP: Total protein
