# Supplementary material for: Analysis of dentin wear and biological properties promoted by experimental inoffice desensitizing materials
Source: BMC Oral Health. 2024 May 25;24:607. doi: 10.1186/s12903-024-04373-9 (PMC11127430; doi:10.1186/s12903-024-04373-9)
Supplement: Supplementary file 1 — Supplementary Material 1 [file 12903_2024_4373_MOESM1_ESM.docx]

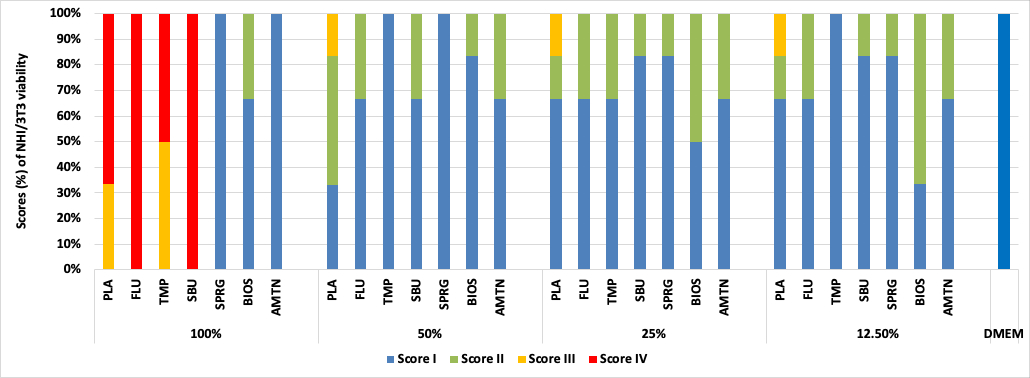


**Figure S1** Categorization of materials (in scores) according to their effect on NHI/3T3 cell viability. Score I: non-cytotoxic (more than 90% cell viability), Score II: slightly cytotoxic (60-90 % cell viability), Score III: moderately cytotoxic (30-59% cell viability); Score IV: severely cytotoxic (less than 30% cell viability) [23]. Placebo varnish (PLA), fluoride varnish (FLU); nanoparticulate sodium trimetaphosphate varnish (TMP); universal adhesive (SBU); surface pre-reacted glass-ionomer filler-containing varnish (SPRG); bioactive ceramic solution (BIOS) and protein from enamel solution (AMTN).


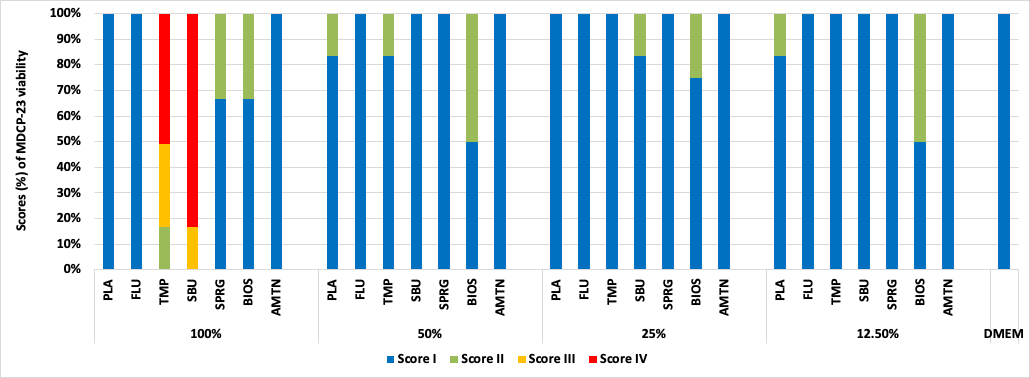


**Figure S2** Categorization of materials (in scores) according to their effect on MDPC-23 cell viability. Score I: non-cytotoxic (more than 90% cell viability), Score II: slightly cytotoxic (60-90 % cell viability), Score III: moderately cytotoxic (30-59% cell viability); Score IV: severely cytotoxic (less than 30% cell viability) [23].

Placebo varnish (PLA), fluoride varnish (FLU); nanoparticulate sodium trimetaphosphate varnish (TMP); universal adhesive (SBU); surface pre-reacted glass-ionomer filler-containing varnish (SPRG); bioactive ceramic solution (BIOS) and protein from enamel solution (AMTN).

**Table S1** Correlations between data from cell viability and total protein analysis obtained for NIH/3T3 cells considering each extract dilution separately (100% - extract, 50% diluted, 25% diluted, 12.5% diluted in DMEM)

|  | **L3T3** | **PLA** | **FLU** | **TMP** | **SBU** | **SPRG** | **BIOS** | **AMTN** | **DMEM** |
| --- | --- | --- | --- | --- | --- | --- | --- | --- | --- |
| **100%** | Spearman | -0.371 | -0.058 | -0.714 | 0.9 | 0.928 | 1 | 0.029 | -0.714 |
|  | p-valor | 0.497 | 0.913 | 0.136 | 0.083 | 0.008 | 0.003 | 1 | 0.068 |
| **50%** | Spearman | 0.348 | -0.257 | 0.257 | -0.086 | 0.371 | 1 | 0.377 |  |
|  | p-valor | 0.499 | 0.658 | 0.658 | 0.919 | 0.497 | 0.003 | 0.461 |  |
| **25%** | Spearman | 0.829 | 0.771 | -0.886 | 0.116 | 1 | 1 | 0.143 |  |
|  | p-valor | 0.058 | 0.103 | 0.033 | 0.827 | 0.003 | 0.003 | 0.803 |  |
| **12.5%** | Spearman | 0.6 | -0.543 | -0.9 | -0.314 | 0.543 | 1 | -0.314 |  |
|  | p-valor | 0.242 | 0.297 | 0.083 | 0.564 | 0.297 | 0.003 | 0.564 |  |

**Table S2** Correlation between data from cell viability and total protein analysis of MDPC-23 cells considering each extract dilution separately (100% - extract, 50% diluted, 25% diluted, 12.5% diluted in DMEM).

|  | **MDPC** | **PLA** | **FLU** | **TMP** | **SBU** | **SPRG** | **BIOS** | **AMTN** | **DMEM** |
| --- | --- | --- | --- | --- | --- | --- | --- | --- | --- |
| **100%** | Spearman | -0.543 | 0.116 | 0.406 | -0.257 | 0.986 | 0.543 | -0.143 | -0.8 |
|  | p-valor | 0.297 | 0.827 | 0.425 | 0.658 | <0.001 | 0.297 | 0.803 | 0.333 |
| **50%** | Spearman | -0.609 | -0.6 | -0.145 | -0.257 | -0.314 | 0.257 | -0.551 |  |
|  | p-valor | 0.2 | 0.242 | 0.784 | 0.658 | 0.564 | 0.658 | 0.257 |  |
| **25%** | Spearman | -0.714 | -0.714 | -0.429 | -0.203 | -0.029 | 0.029 | -0.314 |  |
|  | p-valor | 0.136 | 0.136 | 0.419 | 0.7 | 1 | 1 | 0.564 |  |
| **12.5%** | Spearman | -0.6 | 0.657 | -0.429 | 0.086 | 0.371 | -0.812 | -0.771 |  |
|  | p-valor | 0.242 | 0.175 | 0.419 | 0.919 | 0.497 | 0.05 | 0.103 |  |
